# Seventy-seven days on veno-venous extracorporeal membrane oxygenation for severe acute respiratory distress syndrome from SARS-CoV-2

**DOI:** 10.1177/02676591221103545

**Published:** 2022-06-02

**Authors:** Constantin Shuster, Hussein Kanji, George Isac, Roxanne Jeovens, Amandeep Sidhu, Simmie Kalan, John Yee, Gordon Finlayson

**Affiliations:** 1 Division of Critical Care Medicine, Department of Medicine, 12358 University of British Columbia, Vancouver, BC, Canada; 2 Division of Thoracic Surgery, Department of Surgery, 12358 University of British Columbia, Vancouver, BC, Canada

**Keywords:** Extracorporeal membrane oxygenation, acute respiratory distress syndrome, COVID-19, pulmonary fibrosis, physiotherapy

## Abstract

Throughout the COVID-19 pandemic veno-venous extracorporeal membrane oxygenation (VV ECMO) has emerged as a valid supportive intervention for severe COVID-19 pneumonia. In this report we describe the use of prolonged ECMO (77 days) to support a patient with COVID-19, ultimately resulting in lung recovery and discharge home. This report also emphasizes the value of physiotherapy in patients on ECMO and the importance of collaboration between ECMO programs and lung transplant teams in the care of these patients.

## Case Report

**Figure 1.**
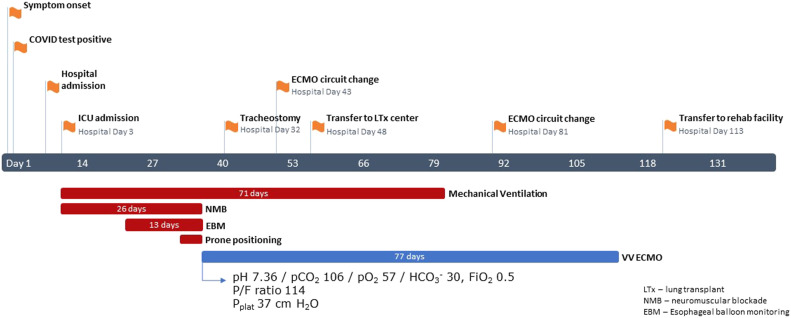
Timeline of the patient’s clinical course including all initiated adjunct supportive interventions for severe ARDS. Ceftriaxone and azithromycin were prescribed on hospital admission with completion of a full course. The patient also completed a 10-day course of dexamethasone at 6 mg PO/IV daily starting on hospital admission. The patient did not receive Tocilizumab as evidence was not published yet. Head of bed was elevated above 30° during mechanical ventilation, except when in the prone position; when prone positioning was employed it was performed for 16 h per day. During mechanical ventilation, S_a_O2 goal was 88–95%, P_a_O2 goal was 55–80 mmHg. While on VV ECMO S_a_O2 goal was >85%; CO_2_ clearance was titrated to target normal pH with no more than a 14 mmHg reduction in CO2 in the first 24 h. The patient was discharged home after the rehab facility.

**Figure 2.**
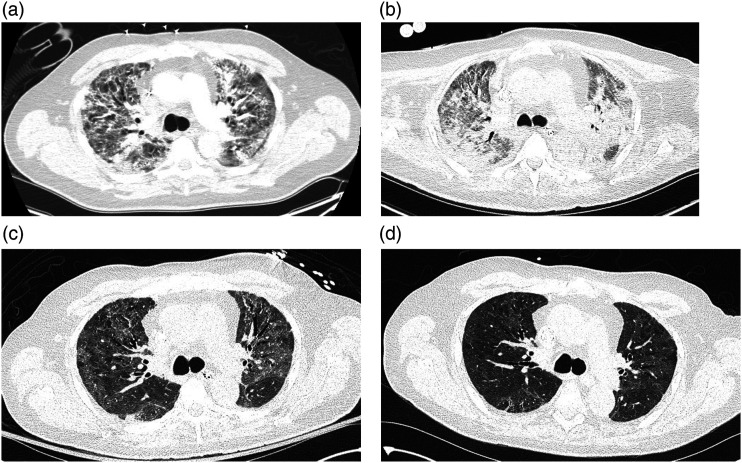
Computed tomography images of the chest at the level of the carina taken on hospital day 21 (a) day 44 (b) day 84 (c) and day 103 (d). Initial scans show diffuse bilateral peribronchovascular consolidation with ground glass opacities. As the disease progressed bilateral upper lobes developed a fibro-reticular pattern consistent with early pulmonary fibrosis. The patient was decannulated on hospital day 105 and discharged to a rehab facility on day 113.

An independent 59-year-old male developed severe COVID-19 pneumonia requiring mechanical ventilation. During his third week of ICU care, despite optimal medical management (Figure 1) and strict adherence to lung protective mechanical ventilation, ventilating pressures became prohibitively high and gas exchange threatened survival (Figure 2(a)). As rescue support, veno-venous (VV) ECMO with bi-femoral cannulation was initiated on hospital day 28.

To facilitate recovery, he received antibiotics for ventilator-associated pneumonia, high dose steroids for organizing pneumonia, continuous dialysis to manage fluid balance and a tracheostomy (day 32). Subsequent imaging (Figure 2(b)) showed worsening lung injury. He was transferred to our provincial ECMO and lung transplant center for assessment (day 48). His circuit was reconfigured to a dual lumen Avalon, enabling participation in physiotherapy.

Upon ICU admission at the transplant center, blood cultures grew *Staphylococcus epidermidis*. Indwelling vascular catheters (except the Avalon) were replaced, the ECMO circuit was exchanged and an extended course of vancomycin was prescribed. Clots on the oxygenator of the second circuit prompted another circuit change. He was listed for transplant following resolution of the bacteremia on day 78.

While awaiting transplant, management goals emphasized physical rehabilitation. Sedation was weaned, assisting liberation from mechanical ventilation. He was then trialed off ECMO (sweep gas interrupted with maintenance of blood flow), breathing only with CPAP support. This clinical junction raised a challenging question - whether lung transplantation would offer improved long-term survival given his improved trajectory? When gas flow was interrupted on the ECMO circuit, there was no meaningful effect on oxygenation, but his native minute ventilation increased markedly (>20 L/min); accordingly, we decided to continue with full ECMO support to facilitate physiotherapy. Over time the ECMO gas flow was weaned during rehabilitation sessions. He was decannulated on hospital day 105 and discharged to a rehab facility 8 days later. Pulmonary function tests on discharge and at 4-month follow-up showed severe restrictive lung disease with ongoing need for supplemental oxygen (Table 1).

**Table 1.** Pulmonary function and 6-minute walk test results.

Parameter	Hospital discharge	4 months post-discharge
TLC	2.41 L (35%)	2.81 L (42%)
FVC	1.49 L (34%)	1.58 L (35%)
FEV1	1.35 L (39%)	1.37 L (39%)
DLCO	10 mL/min/mmHg (38%)	10.7 mL/min/mmHg (38%)
6MWD	200 m	310 m
6MWOS	90% (4 L/min)	93% (increase to 5 L/min)

TLC – Total lung capacity; FVC – Forced vital capacity; FEV1 – Forced expiratory volume in 1 s; DLCO – diffusing capacity of the lungs for carbon monoxide; 6MWD – 6-minute walk distance; 6MWOS – 6-minute walk end oxygen saturation.

## Discussion

This case highlights the possibility of lung recovery after prolonged VV ECMO support (77 days) for hypoxemic respiratory failure from COVID-19 associated acute respiratory distress syndrome (ARDS). Although initially transferred for lung transplant assessment, the team ultimately decided to continue utilizing VV ECMO as a bridge to recovery after witnessing clinical improvement. This decision required ongoing reassessment and collaborative input between the ECMO program and lung transplant team, employing an open decision-making process focusing centrally on the patient’s long-term outcome. The teams constantly weighed the risks and prognosis of lung transplantation against complications of prolonged VV ECMO and the uncertain recovery from COVID-19.

While weaning from ECMO, our priority was to minimize sedation, encourage spontaneous breathing and engage in a rigorous rehabilitation program. ECMO allowed for participation in physiotherapy to recover strength and ambulation (Supplementary Material video). Physiotherapy and rehabilitation for patients supported by VV ECMO has been described as safe, important for ECMO weaning and associated with reduced ICU mortality.^1,2^

Given the degree of lung parenchymal injury, long-term functional recovery remained unclear. To assist prognostication, we monitored the patient’s rehabilitation progress, while on ECMO. Vital signs, central venous oxygen saturation (ScvO2) and lactate levels were assessed before/after sessions while gradually weaning ECMO. Initially, with only 1-2 steps taken, ScvO2 decreased below 50% and lactate levels rose above 6 mmol/L. His initial lack of physiologic reserve significantly improved with ongoing physiotherapy. Serial CT scans were obtained to interrogate radiographic recovery of the lung injury (Figures 2(c) and (d)). Except while actively infected, he remained listed for transplant in case his clinical trajectory plateaued. After mobilizing successfully with ECMO weaned (without significant physiologic restriction), coupled with radiographic improvement, we felt confident in safely and permanently separating from ECMO.

### VV ECMO in ARDS

The use of VV ECMO in severe ARDS has increased worldwide with experience, technological improvements and publications of the CESAR and EOLIA trials.^3,4^ The benefit of VV ECMO in severe ARDS may be derived from reduction of ventilator-induced lung injury.^5^ Initiation, maintenance and weaning from ECMO requires a specialized ICU team with higher volume centres (>30 patients/year) consistently reporting improved outcomes, both before and during the COVID-19 pandemic.^6,7^

### VV ECMO in COVID-19

In the early stages of the pandemic, mortality rates were >50% for critically ill patients.^8^ Initial case series of VV ECMO support for COVID-19 carried a risk of death approaching 100%.^9^ Later reports described improved outcomes for VV ECMO in this patient population.^10^ The largest report was from the ELSO registry which included 1035 patients with COVID-19 across 36 countries.^11^ The estimated 90-day in-hospital mortality was 38% for those on VV ECMO, the median duration between starting mechanical ventilation and ECMO was 4 days, and the median time on ECMO was 13.9 days. These outcomes parallel those published in the EOLIA and CESAR trials.

Our patient’s duration on VV ECMO as a bridge to recovery was significantly longer than had been initially documented in the literature. Recent studies from France and China have documented similar results highlighting the potential of lung recovery.^12–14^ Recent lung transplantation data for COVID-19 have also described prolonged bridging with ECMO support (median of 49 days).^15^ This case highlights that when care is provided by experienced and collaborative teams, prolonged VV ECMO for COVID-19 is feasible and may result in hospital discharge and functional recovery.

Lung recovery in patients with fibrosis from COVID-19 remains unknown; perhaps the patient described herein may require future transplantation. With over 200 million people worldwide afflicted by COVID-19 to date, survivors will need to be monitored to mitigate a wave of late mortality.^16^ Locally, post-COVID-19 clinics have been established where our patient is monitored for disease sequelae.

## Supplemental Material

**Figure:** Video-1
